# Nucleosomal embedding reshapes the dynamics of abasic sites

**DOI:** 10.1038/s41598-020-73997-y

**Published:** 2020-10-14

**Authors:** Emmanuelle Bignon, Victor E. P. Claerbout, Tao Jiang, Christophe Morell, Natacha Gillet, Elise Dumont

**Affiliations:** 1 Univ. Lyon, ENS de Lyon, CNRS UMR 5182, Université Claude Bernard Lyon 1, Laboratoire de Chimie, F69342 Lyon, France; 2 Institut des Sciences Analytiques, UMR 5280, Université de Lyon 1 (UCBL) CNRS, Lyon, France; 3 Institut Universitaire de France, 5 rue Descartes, 75005 Paris, France

**Keywords:** DNA, Theoretical chemistry, Nucleic acids

## Abstract

Apurinic/apyrimidinic (AP) sites are the most common DNA lesions, which benefit from a most efficient repair by the base excision pathway. The impact of losing a nucleobase on the conformation and dynamics of B-DNA is well characterized. Yet AP sites seem to present an entirely different chemistry in nucleosomal DNA, with lifetimes reduced up to 100-fold, and the much increased formation of covalent DNA-protein cross-links leading to strand breaks, refractory to repair. We report microsecond range, all-atom molecular dynamics simulations that capture the conformational dynamics of AP sites and their tetrahydrofuran analogs at two symmetrical positions within a nucleosome core particle, starting from a recent crystal structure. Different behaviours between the deoxyribo-based and tetrahydrofuran-type abasic sites are evidenced. The two solvent-exposed lesion sites present contrasted extrahelicities, revealing the crucial role of the position of a defect around the histone core. Our all-atom simulations also identify and quantify the frequency of several spontaneous, non-covalent interactions between AP and positively-charged residues from the histones H2A and H2B tails that prefigure DNA-protein cross-links. Such an in silico mapping of DNA-protein cross-links gives important insights for further experimental studies involving mutagenesis and truncation of histone tails to unravel mechanisms of DPCs formation.

## Introduction

**Figure 1:**
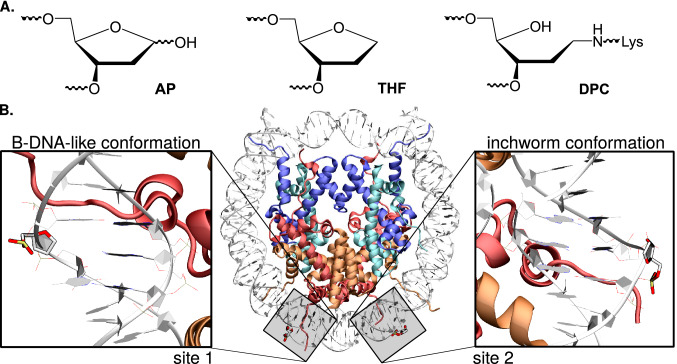
(**A**) Chemical structure of an AP site, its THF analog, and DNA-protein cross-link (DPC) formed upon reaction between a lysine and an AP site. (**B**) Cartoon representation of the crystal structure of a nucleosome core particle harboring two symmetrical solvent-exposed tetrahydrofuran residues (sites 1 and 2; PDB ID 5JRG). Site 1 exhibits a classical B-DNA-like conformation, while site 2 adopts the so-called inchworm conformation following the exclusion of THF and of an adenine on the opposite strand. Biomolecule pictures were rendered with VMD 1.9.4a37 (https://www.ks.uiuc.edu/Research/vmd/)^19,20^. All the figures were rearranged using Inkscape 0.91 (https://inkscape.org/).

Apurinic/apyrimidinic (AP) sites are the most frequent spontaneous DNA lesions, amounting to 5000–10,000 lesions per day per mammal cell^1,2^. They are generated by hydrolysis of the N-glycosylic bond between a nucleobase and a deoxyribose^3^, spontaneously or as intermediates in the excision repair of damaged bases. Their repair by dedicated enzymes turns out to be most efficient by base excision repair (BER) but also nucleotide excision repair (NER)^4^. Yet accumulation of AP sites, notably triggered by exogenous oxidative stress, represents a threat for genome integrity. Notably AP sites can act as precursors towards much more deleterious lesions such as interstrand cross-links, strand breaks and DNA-protein cross-links (DPCs)^5^, through the reactive aldehyde moiety of their open conformation^6^ (see Fig. 1A). Hence understanding AP sites chemistry and DPCs formation is of utmost importance for the development of anticancer therapies^7,8^. Structural and thermodynamic features of AP sites as well as their repair have been intensively studied for oligonucleotides and G-quadruplexes^9–11^: the structural reorganization of the “hole” left within the B-helix by the nucleobase removal and the non-covalent interactions involving the orphan nucleobase on the opposite strand remain to be elucidated. Recent chemical methods have been proposed to sequence them in DNA with single-nucleotide resolution^12^. Yet until recently, insights into the chemistry of AP sites within nucleosomal DNA were scarce. Greenberg and coworkers have reported vastly shortened lifetimes for AP sites embedded within nucleosome core particles (NCPs). A recent X-Ray structure, shown in Fig. 1B, has been obtained by Osakabe and coworkers^13^. This structure features a segment of ds-DNA, comprising 145 base pairs (bp) wrapped onto an octameric core of histone proteins: H2A, H2B, H3 and H4. It harbors two tetrahydrofuran analogs of AP site (THF, see Fig. 1A) at symmetrical superhelical location SHL4.5.

Little is known about in vivo DPCs involving histone tails as they rapidly undergo hydrolysis towards the formation of strand breaks, rendering them rather unstable and difficult to observe. Nevertheless, the use of experimental techniques such as chromatin immunoprecipitation seems promising for their efficient detection in the native chromatin environment^14^. Recently H2B and H2A have been suggested to form Schiff base conjugates with 5-Formylcytosine in vitro and in vivo^15,16^, but there is a lack of data about histone cross-linking with AP sites in vivo. Histones possess short tails which play a significant role, notably their interactions with AP sites give rise to a much enhanced reactivity compared to naked DNA (100-fold more reactive) depending on the exact positioning of the AP site on the NCP^17,18^. Therefore, understanding the molecular mechanisms behind such histone-DNA interactions remains a current scientific challenge.

Molecular dynamics has become a dominant method to shed insights on: structural^21^ and mechanical properties^22^, reactivity and repair of AP sites^23–25^, and effects of clustered AP sites^26,27^. A few all-atom molecular dynamics simulations have been performed on NCPs, undamaged^28–30^, stacked^31^, with double-strand breaks^32^, or harboring acetylated histone tails^33^. As it has become possible to reach the microsecond range for such systems^34^, they can serve as a computational microscope^35^ to probe the conformational and mechanical cross-talk between an abasic site and its N-DNA environment. In this study, microsecond range all-atom simulations were performed to unravel the structural outcome and time evolution of AP-containing and THF-containing NCP, starting from the X-ray structure obtained by Osakabe et al.^13^. A different structural behavior exhibited by AP and THF sites within the NCP is detailed, and interactions with residues of the H2A tail are mapped.

## Results

**Figure 2:**
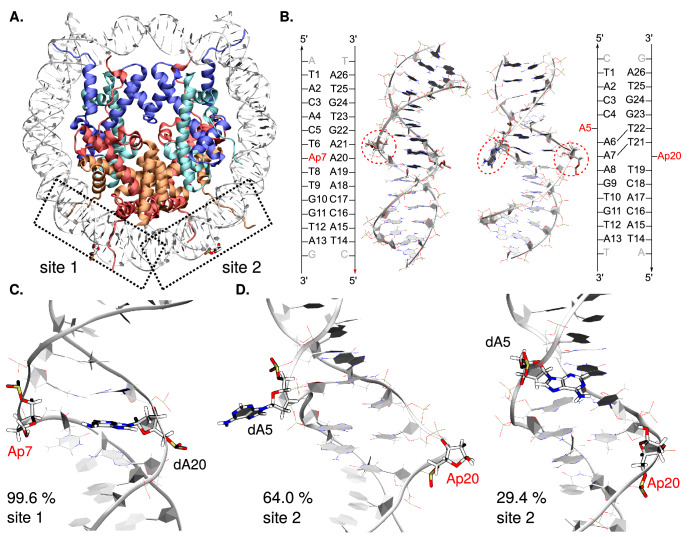
(**A**) Structure of the THF-containing NCP (PDB ID 5JRG^13^). Histones H2A, H2B, H3, and H4 appear in red, orange, blue, and cyan, respectively. The two symmetric lesion sites are depicted in licorice, site 1 on the left (B-DNA like conformation) and site 2 on the right (inchworm conformation). (**B**) Cartoon representation of the NCP 15-bp sections harboring the lesion sites, and their corresponding sequences. The damaged sites (here AP), as well as the in silico modeled dA5 for site 2, are encircled in red. Structural analyses were performed on these 15-bp sections only. Reference MD simulations of naked DNA were performed on the same sequences, with one extra base pair at each extremity to avoid edge effects. (**C**) and (**D**) Representative structures of the main clusters and their frequency of occurrence for site 1 (**C**) and site 2 (**D**) in AP-containing NCP. The lesion sites, the facing base for site 1 (dA20) and the ejected dA5 for site 2 are depicted in licorice. Conformations sampled for site 1 mostly belong to one dominant cluster (99.6% of the simulation time) while site 2 exhibits two main different conformations (64.0% and 29.4% of the simulation time). Pictures were rendered with VMD 1.9.4a37 (https://www.ks.uiuc.edu/Research/vmd/)^19,20^.

The nucleobases numbering used corresponds to the labelling shown in Fig. 2B.

### The two abasic site analogs exhibit contrasted dynamical behavior

Molecular modeling techniques were employed to probe the structural behavior of NCP harboring deoxyribose- and tetrahydrofuran-type abasic sites, hereafter referred to as AP and THF. Three unbiased classical MD simulation replicates of 1 μs each (MD1, MD2, and MD3) were carried out on the NCP structure exhibiting either AP sites or THF analogs at symmetrical superhelical location (SHL) of 4.5, as positioned in the crystal structure by Osakabe et al.^13^. We describe hereafter the local structural features of the two 15-bp sections embedding the damaged sites 1 and 2, as shown in Fig. 2. A first overall measure of the structural impact of an abasic site (or more generally a DNA lesion) on the B-helicity is the bend angle, which was monitored along our MD simulations. For AP sites, the bend angle remains stable around 55 ± 9° in the NCP vs. 27 ± 13° for the control AP-containing sequence (see Figure S1). The bend angle reflects the mechanical constraint exerted along the histone core as the DNA sequence wraps around it^36^, and is found to be rather constant including in the vicinity of AP sites. However the higher curvature may have an impact on the ease to flip out the AP site or a proximal nucleobase compared to naked DNA. This leads us to investigate more locally the structural distortion in the vicinity of the AP sites.

Our simulations allow us to delineate significant differences of local deformation between AP sites. Extrahelicity of AP sites is an important structural signature that can also be monitored along our simulations^24,27^. The extrahelicity criteria is based on the distance between the AP site C1′ atom and its facing nucleotide C1′ atom. The threshold value above which the AP site is considered as extrahelical is 14 Å^22^. The extrahelicity is hereafter expressed as a percentage of time, not necessarily consecutive, during which the lesion is found to be extrahelical in a given simulation. The intrinsic propensity for an AP site to flip out of the B-helix within an oligonucleotide is typically of 30%, as is indeed observed in the control 15-bp sequence simulations. Averaged extrahelicity values reported in Table 1 highlight two very different behaviors for AP sites at site 1 and site 2 within the NCP, with respective values of 19.0% and 88.2%. The NCP environment can induce either a slightly lower extrahelical character at site 1 or, on the contrary, an exacerbated flipping of the lesion at site 2. The initial inchworm conformation of site 2, as well as the ejected dA5 on the facing strand might favor the extrahelical character of the lesion, which is conserved in all our simulations. Noteworthy, the same trend is observed for THF-containing NCP (average average 5.3% site 1 and 85.3% site 2), yet THF exhibits a higher extrahelicity in the control 15-bp (up to 72.8%) suggesting an enhanced lability in naked DNA.

**Table 1:** Averaged extrahelicities over the whole simulation time and detailed values for each 1 μs MD replicate, for AP and THF analogs at site 1 and site 2 in the NCP and in the 1 μs 15-bp control simulations.

System	All (%)	MD1 (%)	MD2 (%)	MD3 (%)	Control 15-bp (%)
AP (Site 1)	19.0	14.4	12.8	29.7	32.1
AP (Site 2)	88.2	67.0	99.6	97.9	39.1
THF (Site 1)	5.3	3.5	1.0	11.5	42.7
THF (Site 2)	85.3	56.6	99.5	99.7	72.8

Experimentally, THF is often invoked as an experimental model for deoxyribo-type AP sites, since this analog offers a higher stability^11,21,37^. Yet it presents chemical and structural differences, with consequences in terms of repair^38^. Simulations for the THF-containing nucleosome were performed to analyze the dynamic behavior of THF within a NCP. Unlike AP sites, THF moieties lack the chemical features to form hydrogen bonds within the double helix. They tend to show a higher extrahelicity than canonical AP sites in the reference 15-bp oligonucleotides but not in the NCP environment—see Table 1.

At site 1 in NCP, THF remains within the double helix, often slightly rotated towards the major groove—Figure S3. No significant reorganization of the surroundings is observed, with the facing dA20 only transiently interacting with dT6 and dT8 flanking THF. Contrary to the control 15-bp, its extrahelicity is found to be very low, i.e. 5.3% in average.

At site 2, the inchworm conformation is well conserved for THF—see Figure S3. dA5 remains ejected and interacts with the H2B tail as seen with deoxyribo-AP—see Figure S5. As dA5 and THF remain ejected from the double helix, a reorganization of the surrounding nucleobases is observed. A strong Watson-Crick pairing and $\pi$-stacking is formed between the adjacent base pairs, as could be found in a standard B-DNA conformation. In MD1, however, THF tends to slightly rotate back within the double helix. As a consequence, its extrahelicity drops to 56.6%. Interestingly, the lower extrahelical character of THF does not favor dA5 rotation back within the helix as can be observed with AP sites—see Figure S3.

In the 15-bp control oligomer, the dA-dT base pairs adjacent to the lesion site tend to swing among each other. It triggers the reorganization of the internal Watson-Crick pairing as the abasic or THF site is ejected and re-inserted within the double helix along the simulations. THF can be more labile than AP with an extrahelicity up to 72.8% against 30–40% for AP, unlike what is observed in the NCP embedding—see Table 1. Interestingly, the inchworm conformation is spontaneously observed for site 1 control sequence harboring AP, with the ejection of an adenine two base-pairs above on the facing strand. For the artificial 145-bp DNA sequence from Osakabe and coworkers was chosen as an alternation of dA-dT and dC-dG tetramers, the reorganisation of the dA-dT pairing around the lesion site stays easy. As such, ejection of one nucleobase on the facing strand, concomitantly to an extrahelical lesion site, allows the stacking of the remaining trimer in a canonical B-DNA shape, as was observed for clustered abasic sites in previous works^21,27^.

### Cluster and PCA analyses of the MD ensembles

Our thorough structural analysis along the MD ensembles reveals a complex structural behavior with several regimes for the AP-sites conformation as shown for main representative clusters of the three MD replicates in Fig. 2. At site 1, a characteristic deformation of the backbone induced by AP sites, so-called bulge, is identified. The cluster analysis shows that the conformational behavior of this site is rather monotonous, with an extrahelicity of the main cluster of the three MD replicates up to 77%, 83%, and 99%, respectively—see Figure S3. The AP site is not involved in stable contacts within the double-helix. It is interacting only transiently with the facing dA20 and the adjacent dT6 and dT8. In MD1 and MD3, structural fluctuations sometimes allow weak interactions between dA20 and dT8.

**Figure 3:**
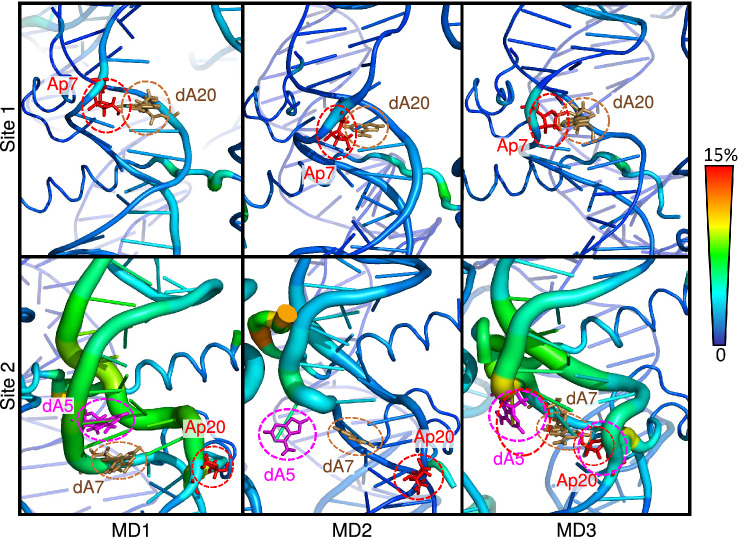
Representation of the per-residue relative contribution to the 10 first principal components around site 1 (top) and site 2 (bottom), detailed for the three MD replicates with AP sites. Only DNA and the N-term tails of H2A and H2B are represented. The percentage of contribution of each residue ranges from 0% (blue) to 15% (red). Abasic sites, its facing orphan base, and the ejected dA5 at site 2 are depicted and encircled in red, magenta and brown, respectively. For a representation of the full NCP, see Figure S6. PCA pictures were rendered using open-source Pymol Version 1.8 (https://pymol.org)^39^.

AP at site 2 exhibits a much more complex structural behavior. One can visualize on Fig. 2D the representative conformations of the two main clusters identified from the simulations. Although the main cluster (observed in 64.0% of the simulation time) shows the AP site in its inchworm conformation, the second one reveals a B-DNA-like structure triggered by the rotation of the initially ejected AP and dA5 back within the double-helix. This is especially observed in the MD1 and MD3 replicates as shown in Figure S3. In MD1, the two most representative clusters (representing 43% and 35% of the simulation time) exhibit a marked return of the ejected adenine to its canonical position within the major groove. Similarly, dA5 starts to flip back within the major groove in the last 100 ns of MD3, corresponding to the third cluster (11% of the simulation time). The latter could be more populated if the trajectories were spanning several microseconds, allowing sufficient time for dA5 to fully retrieve its canonical positioning in the double helix. Interestingly, the extrahelical character of the AP site seems to be positively correlated to the rotation of dA5 out of the helix—see Figure S4. The evolution of the distance between centers of mass of dA5 and its complementary nucleobase (dT22) was chosen as a descriptor of dA5 ejection from the double helix along the trajectory. The threshold distance corresponding to dA5 ejection was fixed at a lower bound of 10 Å by observation of the MD conformational ensembles, the reference distance in canonical B-DNA lying at 6.8 Å—see Figure S4. The evolution of the AP site extrahelicity and dA5 ejection along the MD trajectories indeed shows that the adenine dA5 movement back within the double helix is promoted by a prior decrease of AP extrahelicity. In MD1, the overall extrahelicity of the AP site rapidly decreases in the first 100 ns, allowing dA5 to rapidly flip back within the major groove and form stable canonical interactions until the end of the simulation. In MD3, we observe the same trend in the last 100 ns with the AP site extrahelicity drop concomitant to dA5 rotation towards the major groove—see the third cluster structure in Figure S3. The so-called inchworm conformation of AP at site 2, as found in the crystal structure for THF, remains strongly pronounced in MD2 and in the first 350 ns of MD3 gathering most of the population of cluster 1 (48% of the simulation time).

To overcome the tediousness and potential lack of reproducibility of the visual inspection of the AP sites with the nucleosomal DNA environment, we performed PCA on the whole nucleosome core for each MD (see Fig. 3). The overall picture highlights the localization of salt bridges within the histone core or between histones and DNA (see Figure S6). The motion of the histone tails is also captured. Consistently with our previous observations, the inchworm conformation around site 2 is the most important DNA region with respect to PCs, with a predominance of the ejected dA5 and its neighbours. Its relative importance is higher for MD1 and MD3 where dA5 can flip back in the double helix. On the contrary, the contribution of the surrounding region of site 1 is low in all MD (but slightly higher in MD1). However, the contribution of the abasic site itself is similar in both sites (1.9 to 3.6% of the PC total weight), suggesting that its flexibility is not highly correlated to its initial conformation. These observations on the DNA sites can be correlated with the behaviour of H2A and H2B N-term tails: H2A interacts with the abasic site and does not contribute a lot to PCs whereas the H2B tail, in close contact with dA5 and the inchworm region, present a relatively high mobility. However, the per-residue importance of these tails differs depending on the MD simulation and probably the interaction between its numerous positively-charged residues and the DNA.

### Interaction with histone tails

Beyond the structural distortion of the DNA duplex, the nucleosomal environment offers a high number of residues prone to interact with the phosphate backbone, as well-known, but also and perhaps competitively with the abasic sites and the orphan nucleobase. Our simulations reveal the mobility of the proximal H2A and H2B N-term tails to the damaged sites located at SHL 4.5, as highlighted by the PCA analysis—see Fig. 3. In-depth inspection of histone tail interactions shows that positively-charged lysines and N-term residues are close to damaged sites, prone to react with AP site towards the formation of DNA-protein cross-links.

**Figure 4:**
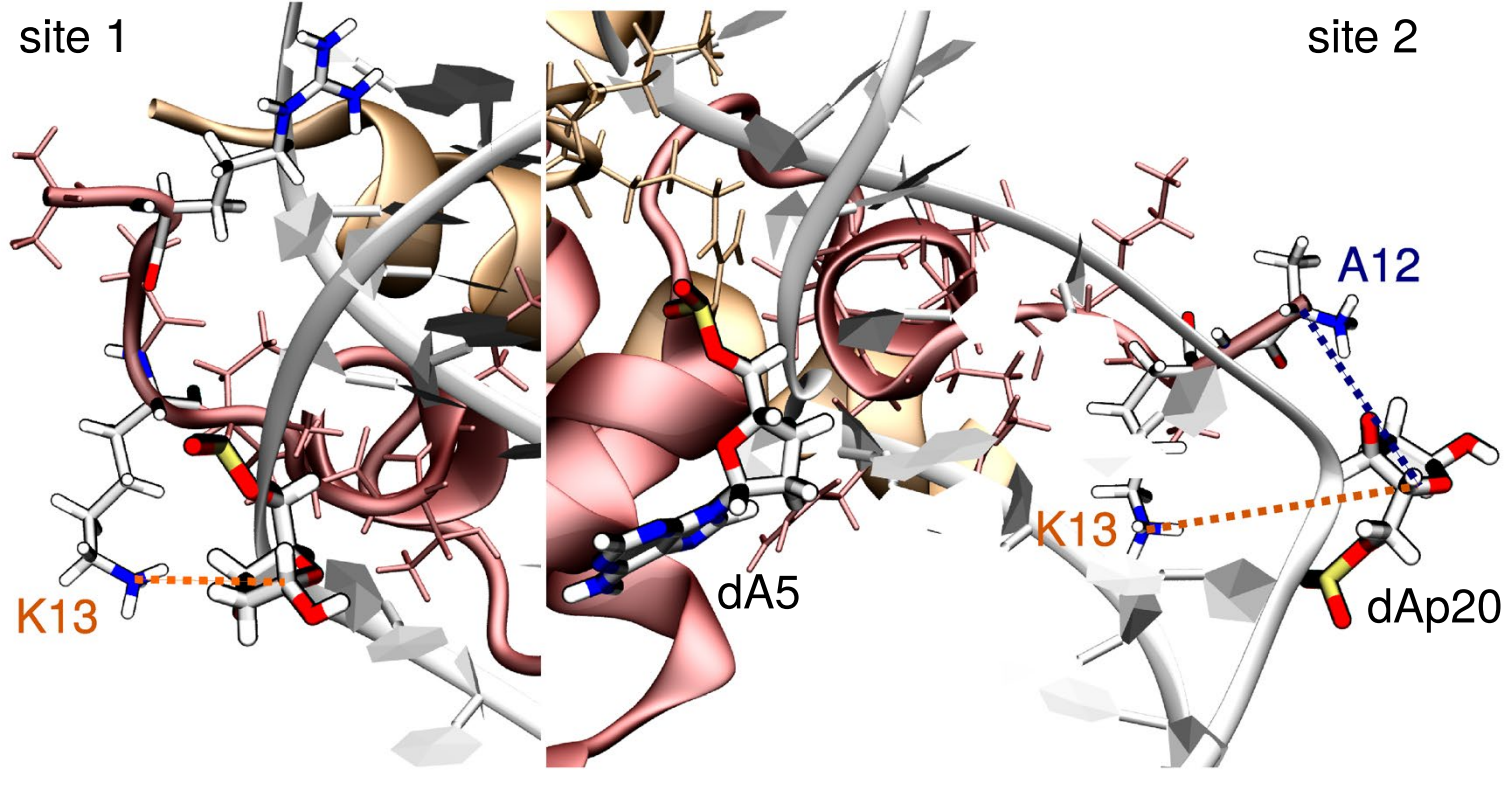
Representation of the main interactions between the damaged site (here AP) and H2A K13 near site 1 (left) and H2A K13 and N-term A12 near site 2 (right). H2B appears in orange and H2A in red, with their tail residues in thin licorice. The abasic sites (dAp7 at site 1 and dAp20 at site 2), the ejected adenine at site 2 (dA5), and the interacting tail residues (K13, N-term A12, and R11) are depicted in thick licorice, colored by element. Pictures were rendered with VMD 1.9.4a37 (https://www.ks.uiuc.edu/Research/vmd/)^19,20^.

Notably, H2A K13 lies within 6 Å of both damaged sites during 6.0–9.2% and 12.2–12.8% of the total simulation time with AP and THF, respectively—see Table 2. Interaction between AP20 and the positively-charged N-term A12 amino acid of the truncated H2A tail is also observed (see Fig. 4), more frequently with the canonical AP site ($\sim$ 20% of the time) than with its THF analog ($\sim$ 7.3% of the time). The H2A tail interacts most of the time in the minor groove nearby both sites, often through insertion of a positively-charged residue, typically R11 two base pairs above site 1 and K13 near site2.

**Table 2:** Frequencies of residence of H2A charged amino groups in a radius lower than to 6 Å of the damaged site.

Interaction	AP (all), %	AP (MD1), %	AP (MD2), %	AP (MD3), %
K13(H2A)-AP7	6.0	4.5	6.1	7.5
K13(H2A)-AP20	9.2	8.3	7.9	11.3
A12(H2A)-AP20	19.9	5.0	30.0	24.8

Interaction	THF (all), %	THF (MD1), %	THF (MD2), %	THF (MD3), %
K13(H2A)-THF7	12.8	1.8	6.2	6.6
K13(H2A)-THF20	12.2	4.8	14.5	17.4
A12(H2A)-THF20	7.3	3.2	10.6	8.2

Percentages are given for AP and THF in the three 1 μs MD replicates (MD1, MD2, MD3) as well as averaged over the replicate (all).

The ejected orphan adenine at site 2 (dA5) also interacts with histone tails. It mainly forms cation-pi interactions with H2B basic residues (K27, K28, R29, R33) and eventually interacts through its amino group with the negatively-charged backbone of the second turn double-helix of DNA—see Figure S5. The nature of the ejected nucleobase might probably not perturb this interaction pattern, for dC, dG and dT can be involved in the same type of contacts as dA. In simulations with AP sites, these interactions fade as the adenine slides back within the DNA helix, which is interestingly not observed with THF. It suggests that the presence of AP sites instead of THF destabilizes the inchworm conformation by favoring the B-DNA-like structure and the reorganization of the interaction network.

## Discussion

In direct line with the recent experimental X-ray investigation^13^, our simulations allow us to test at the microsecond range the stability on the so-called “inchworm” conformation of tetrahydrofuran-type abasic sites. THF sites are confirmed to be stable along our simulations, ruling out a possible crystal packing effect.

A direct comparison between canonical vs. THF-type abasic sites is absent from the literature, especially within a NCP, yet THF analogs are often invoked experimentally to mimic AP sites^11,37^. Our MD investigation allows to “restore” a deoxyribo-type abasic site with exactly the same nucleosomal embedding. It also offers a same-footing comparison with control B-DNA 15-bp oligonucleotides. We observe that the nucleosomal environment reshapes the dynamic behavior of the AP site-bearing double helix with respect to the control naked DNA, by imposing structural constraints through strong interactions with the histone octamer. These structural constraints are reflected in the value of the bend angle around the histone core^40^. The bend angle of the damaged NCP oligonucleotide lies at around 55°, consistent with characteristic value of 41.3° calculated at SHL 4.5 from an undamaged NCP crystal structure (PDB ID 1KX5), much higher than in naked B-DNA—see Figure S2.

Formation of an abasic site leaves a “hole” in the double-stranded helix, which can evolve towards flipping out of the AP site and/or of the orphan nucleobase. The structural outcome of the orphan base, here an adenine, is highly driven by sequence effects^27,41^. The sequence used by Osakabe et al. is a succession of dG-dC and dA-dT tetramers. Consequently, the ejection of the AP-site leads to the rotation of an adenine on the facing strand to allow the stabilization of the tetramer in a canonical trimer-like conformation. This reorganization is observed at site 2 in NCP, but also in the control 15-bp oligomers simulations. This kind of behavior has previously been reported for clustered abasic sites and THF-bearing B-DNA^21,27^.

Inspection of the crystallographic data^13^ suggested that site 1 cannot exhibit an inchworm conformation because of the stretching of the double helix around the histone core induced by the helix shrinking at site 2. In our simulation, site 1 indeed remains in a B-DNA-like conformation. Nevertheless, while THF remains in an inchworm-like conformation at site 2, deoxyribo-AP site shows a different structural behavior. Our simulations reveal that the AP site tends to rotate back within the double helix, concomitantly with the ejected dA5 flipping back to its canonical position. According to our PCA analysis, these motions at site 2 are correlated to an enhanced flexibility at site 1. The propensity of dA5 to flip back within the double-helix appears to be favored by the extrahelicity drop of the facing lesion (see Figure S4), but also to its interaction with the histone core. Within THF-harboring NCP, the ejected adenine interacts strongly with the H2B tail and the backbone of the second turn DNA helix of the NCP (Figure S5), the inchworm conformation remaining conserved in our simulations. On the contrary, these interactions are destabilized upon presence of an AP site instead of THF. The additional hydroxyl group of the AP site might enhance interactions with its surroundings. Therefore, by promoting the classical B-DNA conformation against the inchworm one, the presence of the AP site might trigger the destabilization of ejected dA5 initial interactions to favor its rotation back within the double helix—see Figure S5. As revealed by the PCA analysis, the contribution of the adenine in the overall motion of the system is much more pronounced for the simulation in which site 2 retrieves a classical B-DNA conformation (MD1 and MD3), favored by the disruption of dA5 initial interactions with H2B and the proximal DNA backbone.

Interactions between histone tails and abasic sites are also most important, as they can lead to the formation of DNA-protein cross-links via a Schiff base intermediate between AP and lysines or N-terminal residues, ultimately resulting in deleterious strand breaks. It has been experimentally established that abasic sites and oxidized derivatives undergo rapid strand scission at SHL4.5 and SHL4.7 via DPCs formation with histone tails in vitro^42–44^. H2A and H2B have been reported to be responsible for $\sim$ 77% and $\sim$ 12% of the DPCs with AP sites at SHL4.7, respectively^44^. Interestingly, the basic residues of H2B and H2A tails have also been proposed to react in vitro and in vivo with 5-Formylcytosine^15,16^. Yet the mapping of the amino acids reacting with AP sites at SHL4.5 have not been performed. Our simulations reveal the close proximity of H2A K13 to the damaged site at this position. THF and AP sites are within 6 Å of either K13 or the positively-charged N-term A12 during up to $\sim$ 20% of the simulation time, suggesting the possibility of DPCs formation. Mapping these interactions offers perspectives for experimental studies involving mutagenesis and/or truncation of the H2A tail to assess DPC formation mechanisms and specificity. Moreover, the histone tails are prone to post-translational modifications such as lysine methylation, acetylation, phosphorylation or even ubiquitination which participate to the regulation of the chromatin compaction or the interaction with other proteins (transcription regulator, DNA repair complex)^45^. These modifications can alter the interaction between the damage and the tail^33^, or increase the reactivity of the abasic site^46^ and should also be considered in future works to describe a wider panorama of DPC formations.

Our simulations provide an accurate measure of the contacts occurring between AP sites and positively-charged residues, consistent with in vitro data from the literature. This could be generalized to other nucleosomes, notably more canonical sequences such as $\alpha$-satellite. Indeed the AP-containing nucleosomal structure obtained by X-ray^13^ features a particular repetition of tetramers which was chosen to promote crystallization, but is somehow less representative of canonical N-DNA. Our simulation protocol restores a dynamical view of AP-containing DNA in interaction with a histone core, which remained elusive from the literature. This offers insights at the atomic scale for further experimental studies involving mutagenesis of the histone tails, as it has already been reported for lesions at SHL1.5^47,48^. We are currently using the same protocol to explore the dynamics of a NCP in the presence of clustered abasic sites, to rationalize the drastic increase in DPCs formation rates compared to single AP-sites^49^. MD simulations provide important insights into the finely-tuned sequence effects and dynamics of AP-sites within NCP, which are of utmost important to unravel the complicated mechanisms of their processing by DNA repair enzymes^50^.

## Methods

The crystal structure of a NCP harboring symmetrical THF was used as the starting structure for our models^13^. Two systems were generated from the crystal structure: one with THF as initially in the crystal, the other one with a canonical abasic site. The missing adenine nearby lesion site 2 in the crystal was re-built in silico using xleap. Crystallographic water molecules and chloride ions were kept, and potassium ions were added to neutralize the system. Protonation states of histone core residues were assigned using propka3.1^51^, and histidines were protonated on the $\varepsilon$ position. The two 15-bp sequences harboring each damaged sites (1 and 2) were used to run reference simulations on control naked B-DNA structures with either AP or THF—see Fig. 1. Each system was soaked in a TIP3P water box (truncated octahedron) with a buffer of 10 Å, resulting in $\sim$ 73,500 atoms for NCPs and $\sim$ 25,000–35,000 atoms for the naked 15-bp.

All MD simulations were performed with the Amber and Ambertools 2018 packages^52^. The ff14SB force field was used^53^ together with the OL15 corrections for nucleic acids^54,55^. Force field parameters for non-canonical lesions were generated for THF and taken from previous works for AP sites^24,27^—see Table S1 and Figure S1. The NCP starting structures were carefully optimized through four 10,000 steps energy minimization runs with restraints on the histones and DNA residues, gradually decreased from 20 to 0 kcal mol$^{-1}$ Å$^{2}$. The system was then heated from 0 to 300 K in a 60 ps thermalization run. The temperature was subsequently maintained at 300 K along the simulation using the Langevin thermostat with a collision frequency $\gamma$ln of 1 ps$^{-1}$. The system was then equilibrated during 2 ns in the NPT ensemble. Finally, a one microsecond production run was carried out. For the two NCP systems (with either AP or THF), three simulation replicates (MD1, MD2, MD3) were generated using different starting velocities. The Particle Mesh Ewald method with a 9.0 Å cutoff was used to account for electrostatic interactions. Production runs for the B-DNA reference structures were 1 μs long. Post-processing and structural analyses were carried out using AmberTools 2018 and Curves+^56^ programs. They were performed on the 13-bp section surrounding the damaged sites as showed in Fig. 1. Analyses of the 15-bp DNA reference simulations were consistently ran on the corresponding 13-bp to avoid any edge effects from the floppy first and last base pairs of the oligonucleotide. Clustering of MD ensembles was carried out according to the RMSD of the 13-bp sequence harboring the damaged site showed in Fig. 1B.

The sheer size of the dimensionality of the data from molecular dynamics simulations often makes it difficult for visual inspection of the trajectory, especially for such all-atom N-DNA trajectories. Principal components analysis (PCA) can help reducing the dimensionality by reconstructing a new configurational space that contains the most important degrees of freedom, providing a better angle for grasping the essence of the chemical process. In practice, the PCA method creates a covariance matrix from the coordinates of the trajectory as input. A set of eigenvectors are then obtained by diagonalizing the covariance matrix, serving as basis of a new configurational space, with each of them being a direction of motion. The magnitudes of the eigenvalues indicates the data variance in each of these directions of motions, the eigenvector with the largest eigenvalue is called the first principal component, and so on. The PCA was performed with a home brew script utilizing the Scikit-learn^57^ library, the internal coordinates (inverse distance between geometric centers of two residues) of the trajectory as input instead of Cartesian coordinates, due to better performance^58^. To obtain the per residue importance, the sum of the weighted principal components up to certain threshold with the corresponding as weights is taken. The sum was then normalized and mapped back to the residues.

## Supplementary information

Supplementary Information.
